# Supplementary material for: Epistasis between mutator alleles contributes to germline mutation rate variability in laboratory mice
Source: bioRxiv. 2023 Apr 25:2023.04.25.537217. Preprint. [Version 1] doi: 10.1101/2023.04.25.537217 (PMC10168256; doi:10.1101/2023.04.25.537217)
Supplement: Supplementary file 1 [file media-1.pdf]

Supplementary information

Missense mutations in *Setmar* are unlikely to contribute to epistasis in the BXDs

Unlike *Ogg1* and *Mbd4*, *Setmar* does not participate directly in base-excision repair, though its primate ortholog plays an indirect role in the repair of double-stranded DNA breaks via non-homologous end joining (NHEJ). In anthropoid primates, *SETMAR* encodes a fusion of two functional domains: a SET domain-containing histone methyltransferase and a transposase domain from the *Mariner* family (MAR) [80]; the mouse *Setmar* ortholog only encodes the histone methyltransferase domain. In human cell lines, *SETMAR* localizes to induced double-strand breaks (DSBs) and dimethylates nearby H3K36, which promotes the recruitment of DNA repair components involved in NHEJ to the DSB [35]. There is also evidence that overexpression of *SETMAR* (also known as *Metnase*) improves the efficiency of NHEJ [36] and leads to increased cell survival following exposure to ionizing radiation [36]. Point mutations in either the SET or MAR domains significantly reduced the ability of *SETMAR* to promote non-homologous end joining and DNA repair [36,42,43], suggesting that both domains are needed for its role in DNA repair. Another study found that overexpression of the isolated SET and MAR domains, but not of wild-type *SETMAR*, had a modest effect on NHEJ repair; overexpression of the SET domain slightly *decreased* NHEJ repair of a linearized plasmid in human cells, while overexpression of the *Mariner*-derived domain increased NHEJ relative to controls [81].

Taken together, these results suggest that both the SET and transposase domains of primate *SETMAR* are important for *SETMAR*-mediated DNA repair. The p.Leu103Phe missense mutation that differentiates C57BL/6J and DBA/2J (Table 1) resides within the *Setmar* pre-SET domain and occurs at an amino acid residue that is predicted to be deleterious by SIFT [47]. However, since the mouse *Setmar* ortholog lacks the *Mariner*-derived domain, we believe that the the p.Leu103Phe or p.Ser273Arg missense mutations are unlikely to affect C>A mutation rates in the BXDs. Moreover, we believe that the documented mutator phenotypes associated with both *Ogg1* and *Mbd4*, as well as those genes' known roles in base-excision repair, make them more likely candidates to underlie the epistatic interaction with *Mutyh* we observed in this study.

Supplementary Figures

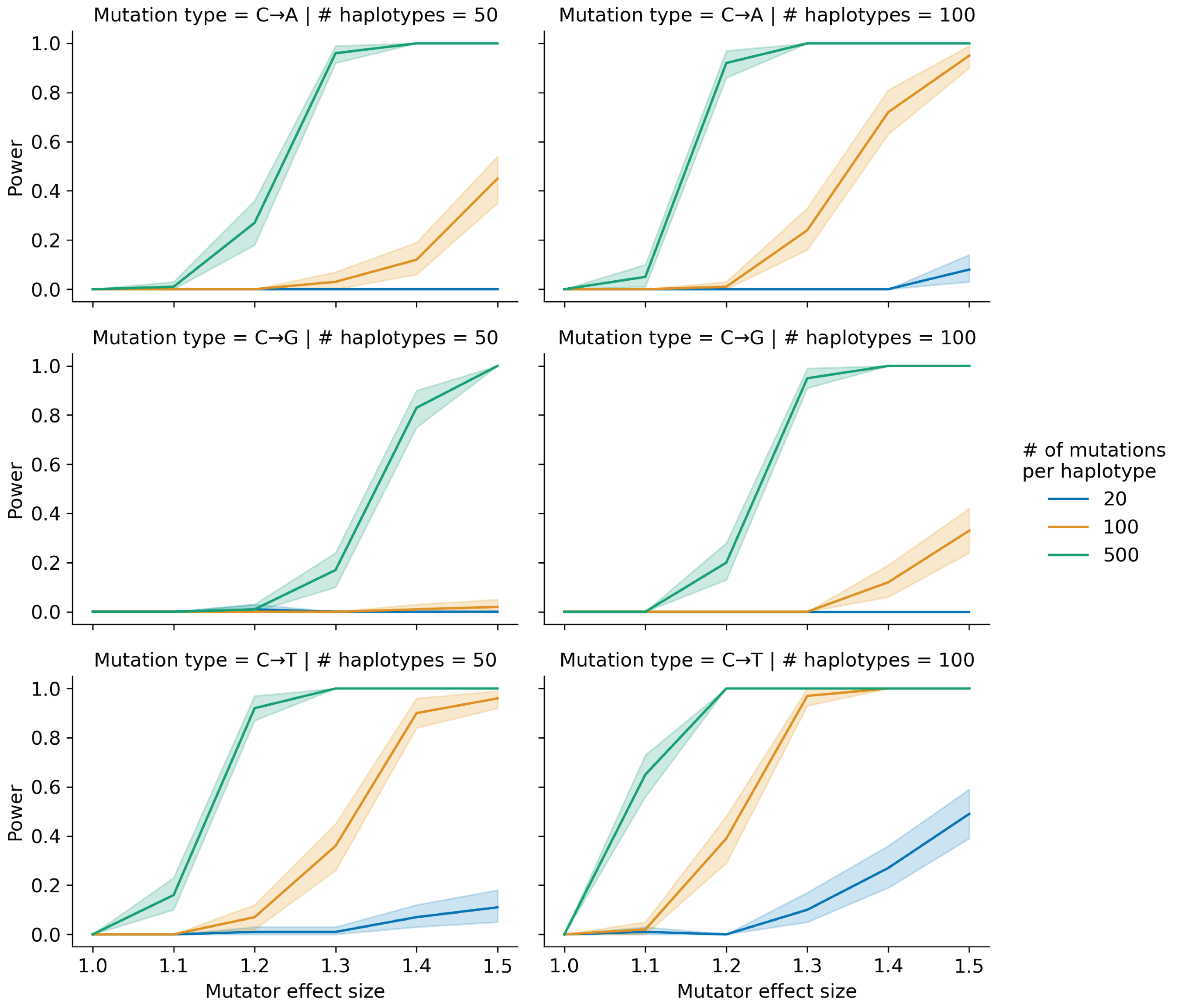

Figure 1-figure supplement 1: Simulations to assess the power of the inter-haplotype distance method. In each of 100 trials, we simulated genotypes at 1,000 biallelic loci on a toy population of either 50 or 100 haplotypes as follows. At every locus on every haplotype, we drew a single floating point value from a uniform distribution $[0, 1)$. If that value was less than or equal to 0.5, we set the allele to be "A"; otherwise, we set the allele to be "B". In each trial, we also simulated *de novo* germline mutations on the population of haplotypes, such that at a single locus g_i, we augmented the mutation rate of a particular k-mer by the specified effect size (an effect size of 1.5 indicates a 50% increase in the mutation rate) on haplotypes carrying "A" alleles. We then applied the inter-haplotype distance method to these simulated data and asked if the adjusted cosine distance at locus g_i was greater than expected by chance. Given a specific combination of parameters, the y-axis denotes the fraction of 100 trials in which the simulated mutator allele could be detected at a significance threshold of p = 0.05. Shaded areas indicate the standard deviation of that fraction across 100 simulations.

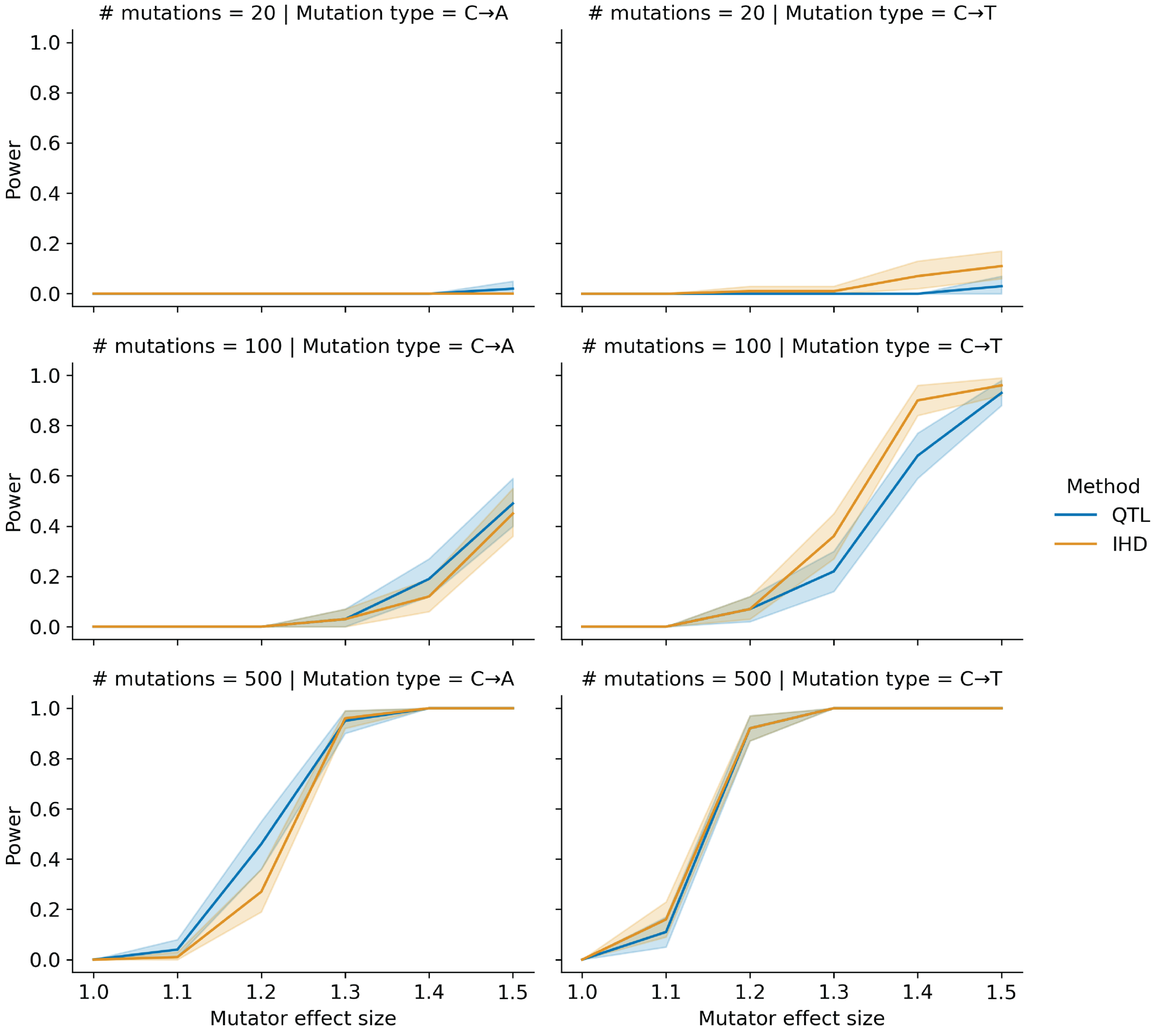

Figure 1-figure supplement 2: Comparing power between the inter-haplotype distance method and QTL mapping. In each of 100 trials, we simulated genotypes at 1,000 biallelic loci on a toy population of 50 haplotypes as follows. At every locus on every haplotype, we drew a single floating point value from a uniform distribution $[0, 1)$. If that value was less than or equal to 0.5, we set the allele to be “A”; otherwise, we set the allele to be “B”. In each trial, we also simulated *de novo* germline mutations on the population of haplotypes, such that at a single locus g_i, we augmented the rate of the specified mutation type by the specified effect size (an effect size of 1.5 indicates a 50% increase in the mutation rate) on haplotypes carrying “A” alleles. We then applied the inter-haplotype distance method to these simulated data and asked if the adjusted cosine distance at locus g_i was greater than expected by chance. Similarly, in each trial, we used R/qtl2 to perform a genome scan for QTL and asked if the log-odds score at g_i was greater than expected by chance. Given a specific combination of parameters, the y-axis denotes the fraction of 100 trials in which the simulated mutator allele could be detected at a significance threshold of p = 0.05 (for IHD) or at an alpha of $\frac{0.05}{7}$ (for QTL mapping). Shaded areas indicate the standard deviation of that fraction across 100 simulations.

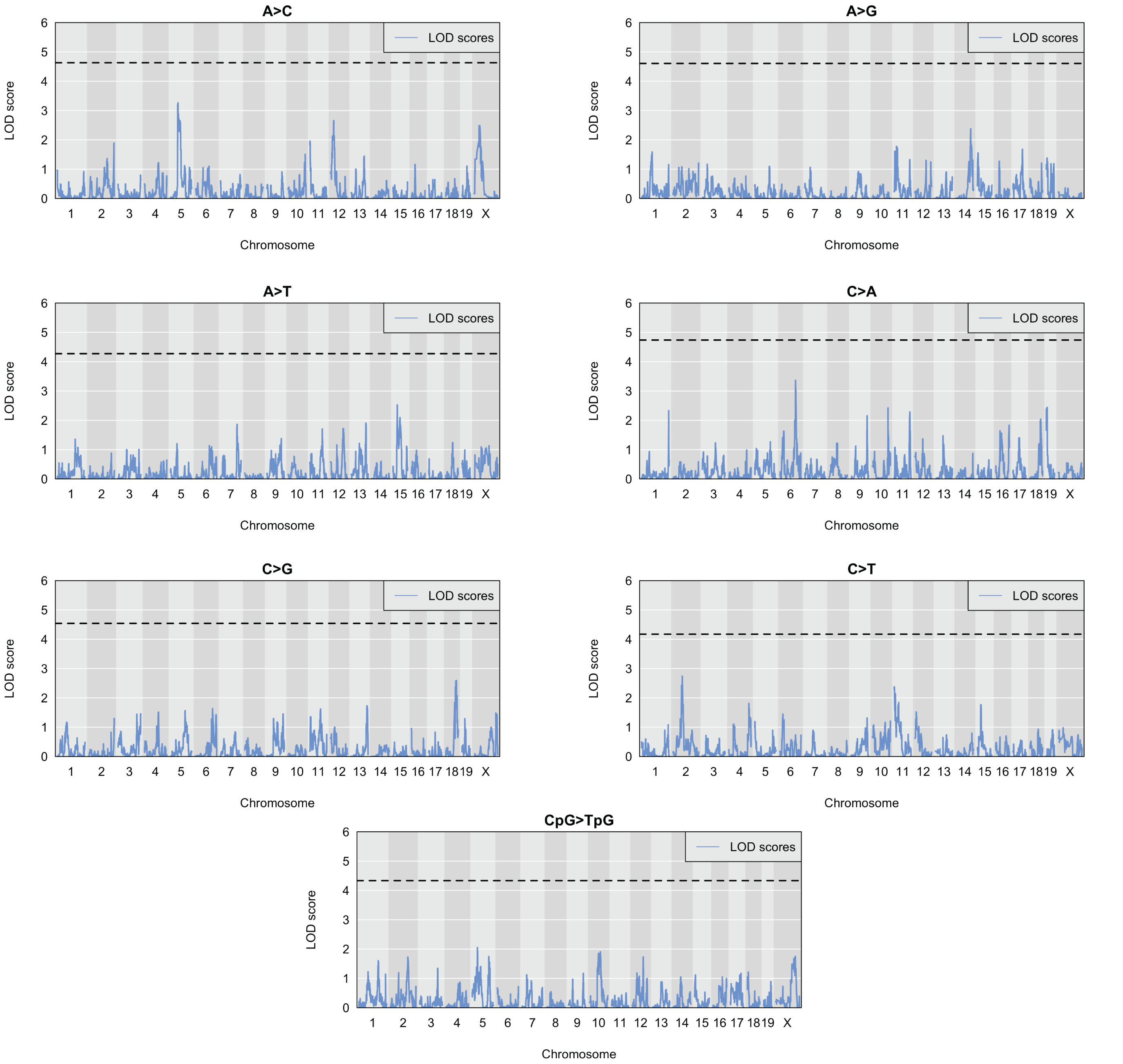

Figure 2-figure supplement 1: Quantitative trait locus scans for mutation spectrum phenotypes. Using the BXDs with D genotypes at `rs27509845` (the marker with the highest cosine distance on chromosome 4; n = 66 BXDs, 42,171 total mutations), we used R/qtl2 to perform QTL scans for the fractions of each 1-mer mutation type. QTL scans also included a kinship matrix (that contained the pairwise genetic similarity between each pair of BXDs, calculated using the leave-one-chromosome-out method) as a random effect term using the `kinship` keyword argument in the `scan1` function. Plots show the log-odds (LOD) score at every genotyped marker in blue; the dotted black line represents the genome-wide LOD significance threshold (established using 1,000 permutations at an alpha of $\frac{0.05}{7}$ to account for the fact that 7 separate association tests were performed.)

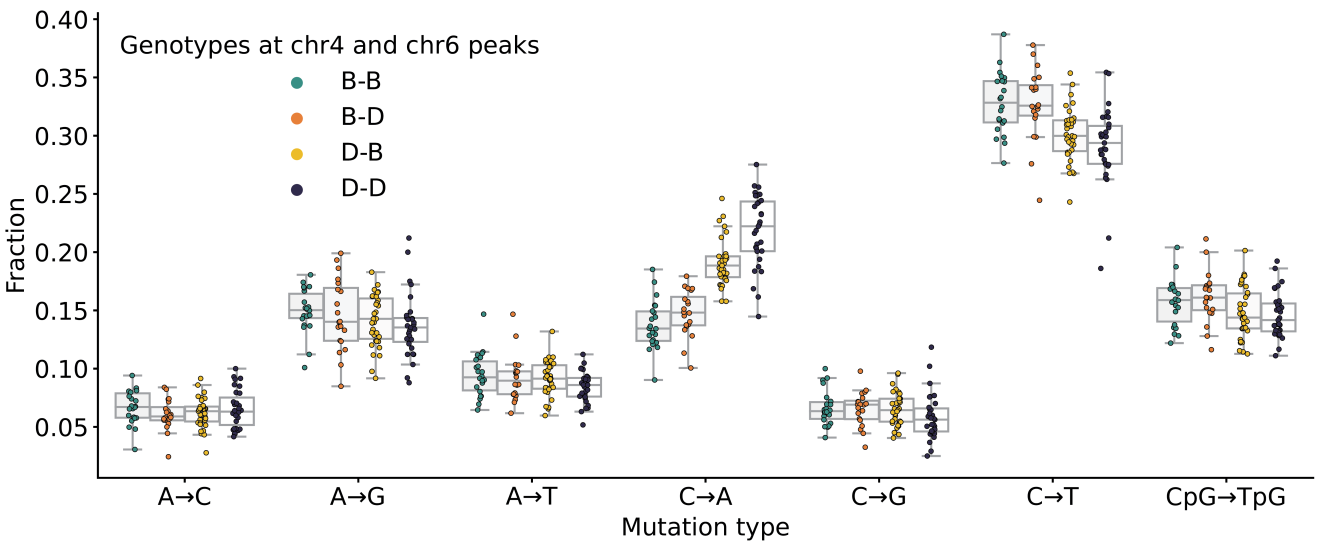

Figure 3-figure supplement 1: Mutation spectra comparison in BXD strains. Fractions of *de novo* germline mutations in BXDs with either *D* or *B* genotypes at markers `rs27509845` and `rs46276051`, stratified by mutation type.

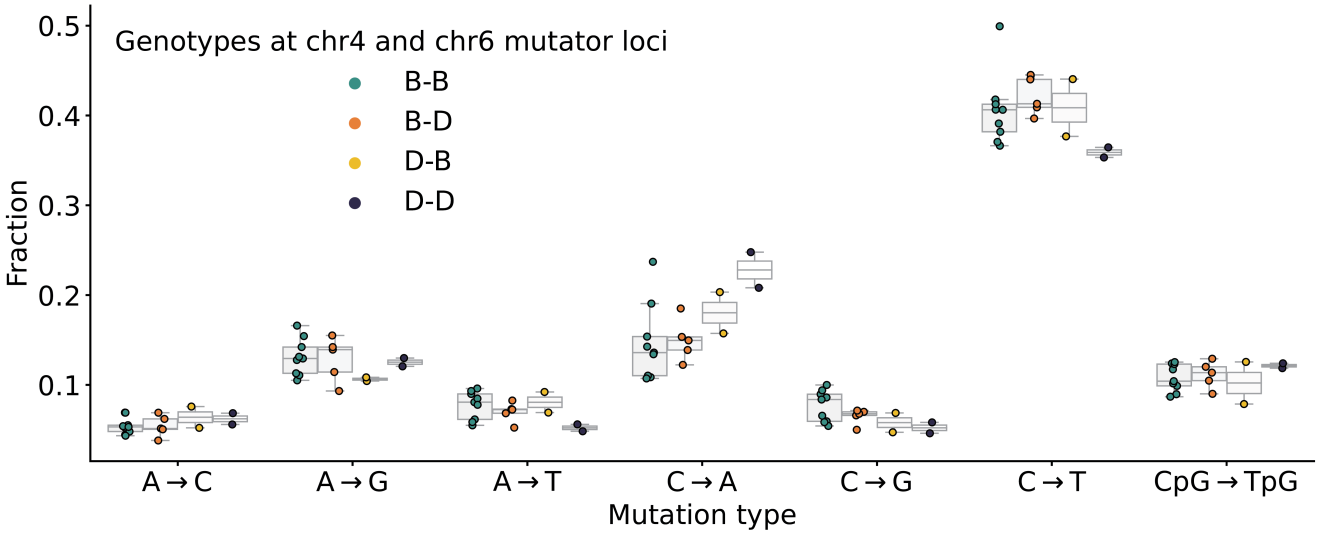

Figure 3—figure supplement 2: Mutation spectra comparison in Sanger Mouse Genomes Project strains. Fractions of *de novo* germline mutations in Sanger MGP strains with either *D* or *B* haplotypes at the chromosome 4 and chromosome 6 mutator loci, stratified by mutation type.

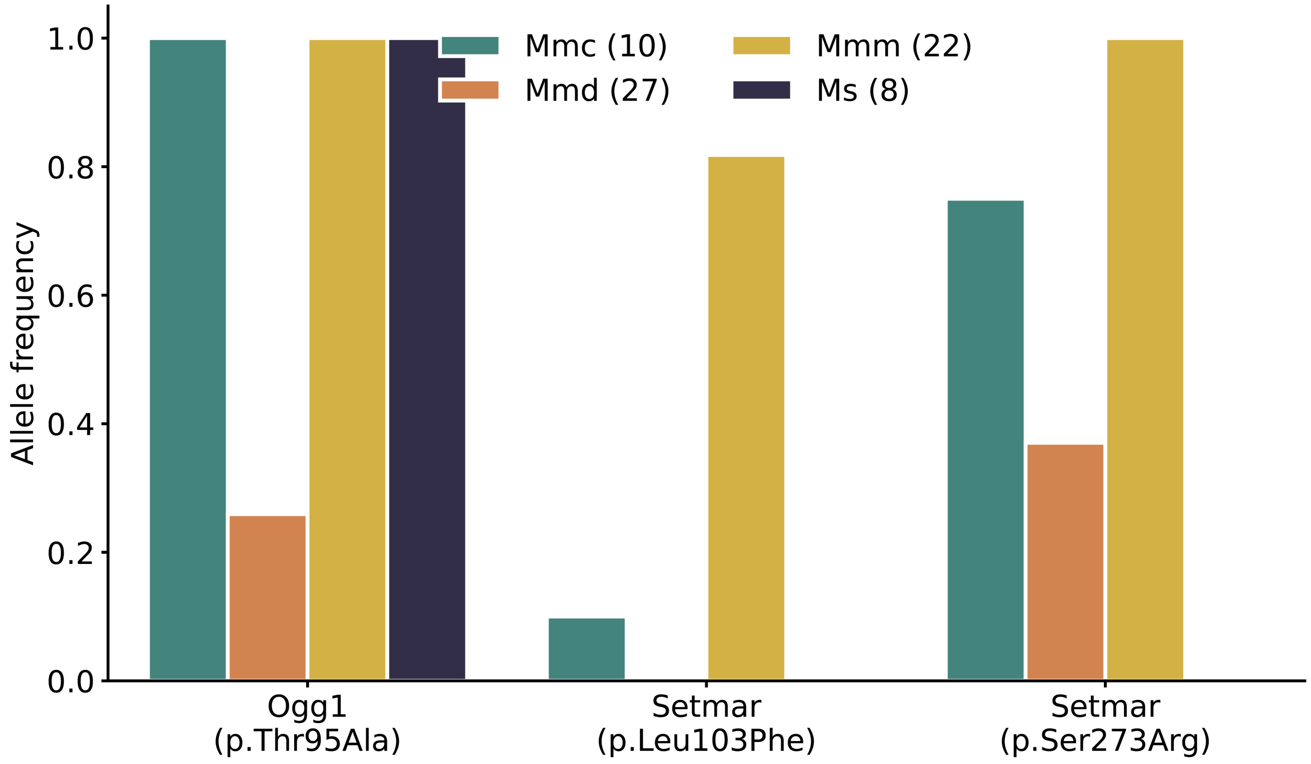

Figure 3-figure supplement 3: Frequency of nonsynonymous DNA repair mutations in wild mice. Alternate allele frequencies of each nonsynonymous DNA repair mutation overlapping the chromosome 6 mutator locus were calculated in populations of wild-derived mice from Harr et al. [30]. Numbers of mice in each subpopulation are shown in parentheses. *Mmc* (*Mus musculus castaneus*), *Mmd* (*Mus musculus domesticus*), *Mmm* (*Mus musculus musculus*), and *Ms* (*Mus spretus*). The *Mbd4* p.Asp129Asn mutation was not observed in any wild populations.

Supplementary Tables

Table supplement 1: Presence or absence of cis-eQTLs for *Ogg1* and *Mbd4* in various tissues identified using GeneNetwork.

Gene name	Tissue name	# BXDs with expression data	Top significant marker	LRS at top significant marker	Additive effect of D allele on expression
Setmar	Kidney	53	-	-	-
Setmar	Gastrointestinal	46	-	-	-
Setmar	Hematopoetic stem cells	22	-	-	-
Setmar	Hematopoetic progenitor cells	23	-	-	-
Setmar	Spleen	79	-	-	-
Setmar	Liver	50	-	-	-
Setmar	Heart	73	-	-	-
Setmar	Eye	87	`rsm10000004172`	56.0	0.151
Ogg1	Kidney	53	`rsm10000004188`	52.25	-0.186

Gene name	Tissue name	# BXDs with expression data	Top significant marker	LRS at top significant marker	Additive effect of D allele on expression
Ogg1	Gastrointestinal	46	`rsm10000003441`	23.39	-0.074
Ogg1	Hematopoetic stem cells	22	-	-	-
Ogg1	Hematopoetic progenitor cells	23	-	-	-
Ogg1	Spleen	79	-	-	-
Ogg1	Liver	50	`rsm10000004188`	53.54	-0.156
Ogg1	Heart	73	-	-	-
Ogg1	Eye	87	`rsm10000004194`	23.05	0.088
Mbd4	Kidney	53	-	-	-
Mbd4	Gastrointestinal	46	-	-	-
Mbd4	Hematopoetic stem cells	22	-	-	-
Mbd4	Hematopoetic progenitor cells	23	-	-	-
Mbd4	Spleen	79	`rsm10000004199`	21.42	0.069
Mbd4	Liver	50	-	-	-
Mbd4	Heart	73	-	-	-
Mbd4	Eye	87	-	-	-
Fancd2	Kidney	53	-	-	-
Fancd2	Gastrointestinal	46	`rsm10000004199`	35.61	0.136
Fancd2	Hematopoetic stem cells	22	-	-	-
Fancd2	Hematopoetic progenitor cells	23	-	-	-
Fancd2	Spleen	79	-	-	-
Fancd2	Liver	50	-	-	-
Fancd2	Heart	73	-	-	-
Fancd2	Eye	87	-	17.79	-0.083
Rad18	Kidney	53	-	-	-
Rad18	Gastrointestinal	46	-	-	-
Rad18	Hematopoetic stem cells	22	-	-	-
Rad18	Hematopoetic progenitor cells	23	-	-	-
Rad18	Spleen	79	-	-	-

Gene name	Tissue name	# BXDs with expression data	Top significant marker	LRS at top significant marker	Additive effect of D allele on expression
Rad18	Liver	50	-	-	-
Rad18	Heart	73	-	-	-
Rad18	Eye	87	`rs252954368`	49.41	-0.131

Table supplement 2: Large structural variants overlapping protein-coding genes in the mutator locus on chromosome 6 All coordinates are with respect to GRCm39/mm39.

SV start	SV end	SV type	Gene name(s)	Overlaps exon?
108,437,600	108,437,652	DEL	*Gm35165*, *Itpr1*	Yes
110,617,754	110,618,347	DEL	*Gm20387*	Yes
113,110,214	113,110,215	INS	*Setd5*	Yes
116,217,366	116,217,367	INS	*Gm52873*, *Washc2*	Yes
112,605,594	112,605,595	INS	*Rad18*	No
112,629,618	112,636,619	DEL	*Rad18*	No
115,823,860	115,824,076	DEL	*Mbd4*	No
